# The use of urinary biomarkers as prognostic tools: predicting kidney outcomes in pediatric acute kidney injury

**DOI:** 10.1007/s00467-025-06920-0

**Published:** 2025-08-13

**Authors:** Wun Fung Hui, Renee Wan Yi Chan, Man Fung Tang, Tony Chun Hei Lei, Tsz Ki Liu, Kwok Hei Ho, Shu Wing Ku, Ting Fan Leung, Kam Lun Hon

**Affiliations:** 1 Department of Paediatrics and Adolescent Medicine, Hong Kong Children’s Hospital, 9/F, Tower B, 1 Shing Cheong Road, Kowloon Bay, Kowloon, Hong Kong; 2 Department of Paediatrics, Faculty of Medicine, The Chinese University of Hong Kong, Hong Kong, Hong Kong; 3 Hong Kong Hub of Paediatric Excellence, The Chinese University of Hong Kong, Hong Kong, Hong Kong; 4 Faculty of Medicine, Laboratory for Paediatric Respiratory Research, Li Ka Shing Institute of Health Sciences, The Chinese University of Hong Kong, Hong Kong, Hong Kong; 5 CUHK-UMCU Joint Research Laboratory of Respiratory Virus & Immunobiology, Department of Paediatrics, Faculty of Medicine, The Chinese University of Hong Kong, Hong Kong, Hong Kong

**Keywords:** Acute kidney injury, Acute kidney disease, Persistent acute kidney injury, Urinary biomarkers, Prognosis

## Abstract

**Background:**

There is limited data on applying urinary biomarkers for prediction of kidney outcomes in pediatric acute kidney injury (AKI).

**Methods:**

We prospectively measured urinary neutrophil gelatinase-associated lipocalin (NGAL), tissue metalloproteinases-2 (TIMP-2), insulin-like growth factor-binding protein 7 (IGFBP-7) and C–C motif chemokine ligand 14 (CCL14), alongside serum kidney function test in critically ill children with AKI admitted to the pediatric intensive care unit. The primary outcomes included persistent AKI (lasting for ≥ 72 h) and prolonged AKI (lasting for ≥ 7 days).

**Results:**

There were altogether 134 patients (median age 4.3 years; 43.3% female; AKI severity stage 1: 44.8%, stage 2: 33.6% and stage 3: 21.6%). The incidence of persistent and prolonged AKI was 40.3% and 25.4%, respectively. All four biomarkers, either measured singly, simultaneously or serially, significantly predicted both outcomes, with NGAL demonstrating the best performance (areas under the curve [AUC] 0.72 [0.61, 0.83] for persistent AKI and 0.72 [0.61, 0.84] for prolonged AKI). Integrating the simultaneous AKI staging with biomarker levels significantly improved prediction (NGAL: AUC 0.86 [0.78, 0.94] for persistent AKI and 0.87 [0.79, 0.96] for prolonged AKI). Persistent AKI increased the risk of acute kidney disease (hazard ratios [HR]: 2.59 [1.55, 4.34]), which was associated with kidney function non-recovery 90 days after AKI (HR 7.73 [1.01, 59.03]).

**Conclusions:**

Urinary NGAL, TIMP-2, IGFBP-7 and CCL14 demonstrated promising performance of predicting kidney function non-recovery within 7 days of AKI onset. Integrating urinary biomarkers with concurrent clinical data substantially enhanced predictive performance.

**Graphical Abstract:**

**Figure:**
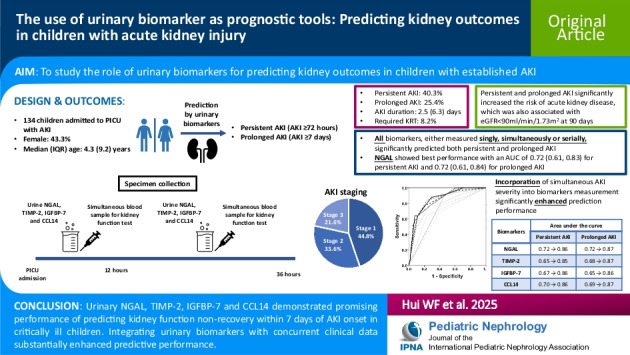
A higher resolution version of the Graphical abstract is available as Supplementary information

**Supplementary Information:**

The online version contains supplementary material available at 10.1007/s00467-025-06920-0.

## Introduction

Acute kidney injury (AKI) is a serious condition affecting 24% to 42% of pediatric intensive care unit (PICU) admissions [1–5]. Numerous studies have revealed its independent impact on PICU mortality and association with adverse kidney outcomes [1, 4–6]. In addition, the trajectory of kidney function after initial AKI also carries significant prognostic weight. Two distinctive entities, namely persistent AKI defined as AKI lasting for more than 72 h [7] and acute kidney disease (AKD) defined as extension of AKI from 7 to 90 days [8], are particularly concerning as both pose an increased risk of chronic kidney disease (CKD) and mortality among AKI survivors [4, 9, 10].

Accurate prediction of kidney outcomes is crucial for guiding post-AKI care. Hence, the role of biomarkers in predicting the trajectories of kidney function has garnered considerable attention. Previous studies indicated that urinary neutrophil gelatinase-associated lipocalin (NGAL), tissue metalloproteinases-2 (TIMP-2), insulin-like growth factor-binding protein 7 (IGFBP-7) and C–C motif chemokine ligand 14 (CCL14) showed promising potential for predicting kidney outcomes among critically ill patients with AKI [11, 12].

NGAL is an early tubular injury marker, while TIMP-2 and IGFBP-7 are cell cycle arrest markers indicating tubular stress [13, 14]. Both have been extensively evaluated in critical care settings [14, 15]. CCL14, also known as human CC chemokine-1, is a chemokine that is responsible for recruitment of macrophages and monocytes in various diseases and is thought to trigger inflammatory processes in injured epithelial cells [11, 16–18]. Its potential for identifying persistent AKI in critically ill adults was first identified in the RUBY study [11].

However, there are limited data evaluating the performance of urinary biomarkers in established pediatric acute kidney injury. This study aimed to investigate the ability of urinary biomarkers to predict kidney outcomes in this population. By integrating biomarker measurements with clinical parameters, we aimed to identify a strategy directly applicable to critically ill children who developed AKI.

## Methodology

This was a prospective study conducted in the PICU of the Hong Kong Children’s Hospital from May 2022 to December 2023. We enrolled children aged ≥ 1 month to < 18 years at the time of PICU admission. Only children who developed AKI were included in the current analysis. The exclusion criteria included pre-existing CKD or impaired kidney function for ≥ 3 months, immediate admission after kidney transplant, or children with no blood sampling during their PICU stay. Children without a urinary catheter were excluded from urine collection.

AKI development was assessed daily using the serum creatinine (SCr) criteria of KDIGO classification [19] (Supplemental Table 1). The baseline SCr level was determined by using the lowest SCr level within the past year and was back-calculated assuming an age-specific normal estimated glomerular filtration rate (eGFR) [20] by validated formulas if it was not available [21, 22]. Serum kidney function was tested in all participants upon PICU admission and at least every 24 h thereafter until PICU discharge. Additional sampling was performed otherwise at the clinical team’s discretion.

We aimed to collect two consecutive urine samples at an interval of 12–24 h from each eligible child to measure levels of NGAL, TIMP-2, IGFBP-7 and CCL14. The first sample was collected within 12 h of PICU admission. All urinary samples were stored at 4 °C, and those stored ≥ 24 h were discarded. Samples were then centrifuged (2000 rpm, 10 min, room temperature) and the supernatant was aliquoted and frozen at − 80 °C until measurement. Biomarkers were measured using commercial enzyme-linked immunosorbent assays (ELISA) according to manufacturers’ recommendations (NGAL and TIMP-2: R&D Systems, Minneapolis, MN, USA; IGFBP-7: Boster Bio, Pleasanton, CA, USA; CCL14: Abcam, Cambridge, UK). A paired serum kidney function was measured concurrently with each urine sample collected.

Clinical and laboratory data were retrieved from our hospital’s electronic database. The concurrent SCr level measured at the time of urine collection was defined as the “simultaneous SCr level”. If a simultaneous measurement was unavailable, the SCr level closest to the time of urine collection within a 24-h window was then recorded. Day 1 was defined as the first day of AKI diagnosis based on the SCr trend. AKI onset within 12 h pre- or post-PICU admission was regarded as “AKI on admission”. Severe AKI was defined as stage 2 AKI or above. Persistent AKI was defined as AKI lasting consecutively for ≥ 72 h, and AKI that did not recover within 7 days (lasted for ≥ 7 days) was regarded as prolonged AKI. AKD was defined as fulfilling the KDIGO SCr criteria from 7 to 90 days post-AKI diagnosis. AKI and AKD duration denoted the total time fulfilling each diagnosis. AKI recovery referred to SCr returning to < 1.5 times of baseline, with recovery time denoting the duration from peak SCr to SCr < 1.5 times of baseline. We also recorded the first SCr level 90 days after acquiring AKI. The primary outcomes were persistent AKI and prolonged AKI, while secondary outcomes included AKD and 90-day eGFR < 90 ml/min/1.73 m^2^.

The study complied with the Declaration of Helsinki and was conducted in accordance with the approved protocol of the research ethics committee of the Hong Kong Children’s Hospital (HKCH-REC-2021–053). Informed written consent was obtained from the parents of participants.

## Statistical analysis

Continuous variables are presented as median (25^th^–75^th^ percentile), while categorical data are reported as number (percentage). The incidences of persistent AKI and prolonged AKI were determined by the proportion of patients fulfilling the respective diagnostic definition relative to the total number of eligible children. AKD incidence was determined among children surviving ≥ 7 days post-AKI. To compare the clinical features and urinary biomarker levels between patients with and without persistent AKI and prolonged AKI, either Student’s *t* test, Mann–Whitney *U* test, chi-squared test or Fisher’s exact test were used as appropriate. Receiver operating characteristic (ROC) curves were constructed to assess the diagnostic efficacy of urinary biomarkers for persistent AKI and prolonged AKI using the area-under-curve (AUC) values. The Youden index was used to determine the optimal threshold level. We also determined the AUCs and relative risk of developing either persistent AKI or prolonged AKI using the following models: single biomarker positivity, combined simultaneous AKI staging and biomarker positivity, multiple biomarkers measured simultaneously and serial biomarker measurements. We employed the Cox proportional hazard model to determine the hazard ratios (HRs) of potential factors responsible for development of AKD. Kaplan–Meier curve was constructed, and log-rank test was used to assess the development of eGFR < 90 ml/min/1.73 m^2^ at 90 days between patients with and without AKD. For 90-day outcomes, patients were censored either at mortality or first kidney function test taken at 90 days after AKI. Statistical significance was defined as *p* < 0.05. The Statistical Package for the Social Sciences (Windows V.23.0; SPSS, Chicago, Illinois, USA) was used for statistical analysis.

## Results

The final analysis included 134 participants (Supplemental Fig. 1). The median (25^th^, 75^th^ percentile) age was 4.3 (1.1, 10.3) years and 43.3% of them were female. Table 1 shows the baseline demographics and clinical features. Severe AKI developed in 55.2% of children, and the median AKI duration increased with disease severity (stage 1: 0.9 days vs. stage 2: 2.7 days vs. stage 3: 7.0 days, *p* < 0.001). The incidence of AKI recovery was lower in those with more severe AKI (stage 1: 90.0% vs. stage 2: 71.1% vs. stage 3: 48.3%, *p* < 0.001). The incidence of persistent AKI was 40.3%, and prolonged AKI was 25.4%. 60.2% of children developed AKD and 22.5% had eGFR < 90 ml/min/1.73 m^2^ by 90 days.

**Table 1:** Clinical characteristics of study participants

Variable			Descriptive statistics (*n* = 134)
Demographic data and clinical features			
Age (year)			4.3 (1.1–10.3)
Female sex			58 (43.3%)
History of cancer			29 (21.6%)
BMT recipient			4 (3.0%)
Post-cardiac operation			54 (40.3%)
Indication of PICU admission	Cardiac*		76 (56.7%)
	Respiratory		9 (6.7%)
	Neurological		2 (1.5%)
	Renal		6 (4.5%)
	Metabolic		1 (0.7%)
	Postoperative care (non-cardiac)#		31 (23.1%)
	Observation of suspected condition		9 (6.7%)
Required invasive ventilation			76 (56.7%)
Duration of invasive ventilation (day)			2.8 (0.8, 5.6)
Required non-invasive ventilation			46 (34.3%)
Duration of non-invasive ventilation (day)			3.0 (1.0, 6.7)
Required inotropes			69 (51.5%)
Duration of inotropic use (day)			3.1 (1.8, 5.9)
PIM3 score predicted mortality (%)			0.9 (0.5–1.9)
Baseline eGFR (ml/min/1.73 m^2^)			150.3 (119.4–205.4)
AKI characteristics			
AKI on admission			111 (82.8%)
AKI staging at diagnosis		Stage 1	90 (67.2%)
		Stage 2	35 (26.1%)
		Stage 3	9 (6.7%)
Worst AKI staging		Stage 1	60 (44.8%)
		Stage 2	45 (33.6%)
		Stage 3	29 (21.6%)
Any requirement of KRT			11 (8.2%)
AKI duration (day)			2.5 (0.9–7.0)
Persistent AKI			54 (40.3%)
Prolonged AKI			34 (25.4%)

Expressed as median (25^th^–75^th^ percentile) or number (percentage)

*Including 54 admissions for post-cardiac surgery

#Including limb reconstructive surgery, urological intervention, gastrostomy insertion and fundoplication, small bowel or large bowel surgery, nasopharyngeal surgery, broncho-laryngeal intervention, cleft palate surgery, choledochal cyst excision and Kasai procedure, hepatic surgery, neurosurgical intervention and thoracic surgery

*AKI* acute kidney injury, *BMT* bone marrow transplantation, *eGFR* estimated glomerular filtration rate, *KRT* kidney replacement therapy, *PIM3* pediatric index of mortality 3

Table 2 shows the comparison of the clinical features between children with and without persistent and prolonged AKI. Both conditions were associated with more severe AKI and the duration of AKI was also significantly longer in persistent AKI. Both conditions significantly increased the risk of AKD (Supplemental Table 2), and children with AKD had a significantly higher risk of developing eGFR < 90 ml/min/1.73 m^2^ 90 days after AKI compared to those without AKD (Supplemental Fig. 2).

**Table 2:** Comparison of clinical features and outcomes between children with different AKI entities

Clinical features		No persistent AKI (*n* = 80)	Persistent AKI (*n* = 54)	*p*-value	No prolonged AKI (*n* = 100)	Prolonged AKI (*n* = 34)	*p*-value
Age (year)		3.4 (0.8-7.0)	5.3 (1.2-11.8)	0.069	3.6 (0.8-9.9)	5.0 (1.5-10.4)	0.243
Female sex		32 (40.0%)	26 (48.1%)	0.350	43 (43.0%)	15 (44.1%)	0.910
History of cancer		12 (15.0%)	17 (31.5%)	**0.023**	18 (18.0%)	11 (32.4%)	0.079
Post-cardiac operation		39 (48.8%)	15 (27.8%)	**0.015**	47 (47.0%)	7 (20.6%)	**0.007**
BMT recipient		3 (3.8%)	1 (1.9%)	0.648	3 (3.0%)	1 (2.9%)	1.000
Required invasive ventilation		50 (62.5%)	26 (48.1%)	0.100	60 (60.0%)	16 (47.1%)	0.188
Required non-invasive ventilation		26 (32.5%)	20 (37.7%)	0.534	37 (37.0%)	9 (27.3%)	0.308
Required inotropes		43 (53.8%)	26 (48.1%)	0.525	56 (56.0%)	13 (38.2%)	0.073
PIM3 score predicted mortality (%)		0.8 (0.4–1.3)	1.2 (0.6–3.2)	0.061	0.9 (0.5–1.5)	1.1 (0.5–2.5)	0.345
Baseline eGFR (ml/min/1.73 m^2^)		133.3 (113.5–188.0)	181.6 (137.2–235.9)	**0.001**	138.6 (113.5–197.8)	184.1 (150.3–248.4)	** < 0.001**
Any AKI on admission		65 (81.2%)	46 (85.2%)	0.553	83 (83.0%)	28 (82.4%)	0.931
AKI staging at diagnosis	Stage 1	61 (76.2%)	29 (53.7%)	**0.009**	73 (73.0%)	17 (50.0%)	**0.020**
	Stage 2	17 (21.2%)	18 (33.3%)		23 (23.0%)	12 (35.3%)	
	Stage 3	2 (2.5%)	7 (13.0%)		4 (4.0%)	5	
Worst AKI staging	Stage 1	49 (61.3%)	11 (20.4%)	** < 0.001**	54 (54.0%)	6 (17.6%)	** < 0.001**
	Stage 2	26 (32.5%)	19 (35.2%)		32 (32.0%)	13 (38.2%)	
	Stage 3	5 (6.2%)	24 (44.4%)		14 (14.0%)	15 (44.1%)	
Any requirement of KRT		5 (6.2%)	6 (11.1%)	0.315	7 (7.0%)	4 (11.8%)	0.469
AKI recovery		79 (98.8%)	21 (38.9%)	** < 0.001**	100 (100%)	0 (0%)	** < 0.001**
Developed AKD (***n*** = 113)		27 (42.2%)	41 (83.7%)	** < 0.001**	38 (45.8%)	30 (100%)	** < 0.001**
Worst AKD staging (***n*** = 113)	Nil	37 (57.8%)	8 (16.3%)	** < 0.001**	45 (54.2%)	0 (0%)	** < 0.001**
	Stage 1	15 (23.4%)	11 (22.4%)		22 (26.5%)	4 (13.3%)	
	Stage 2	8 (12.5%)	13 (26.5%)		13 (15.7%)	8 (26.7%)	
	Stage 3	4 (6.2%)	17 (34.7%)		3 (3.6%)	18 (60.0%)	
AKD duration (day)		17.0 (3.0–34.5)	40.7 (6.9–90.0)	**0.008**	17.0 (3.2–39.0)	43.9 (7.2–90.0)	**0.010**
PICU length of stay (day)		3.1 (1.9–7.9)	4.4 (1.9–10.4)	0.379	4.0 (2.0–8.1)	3.8 (1.1–11.1)	0.874
PICU mortality		0 (0%)	3 (18.8%)	0.526	1 (9.1%)	2 (16.7%)	1.000

Expressed in median (25^th^–75^th^ percentile) or number (percentage)

*AKD* acute kidney disease, *AKI* acute kidney injury, *BMT* bone marrow transplantation, *eGFR* estimated glomerular filtration rate, *KRT* kidney replacement therapy, *PIM3* pediatric index of mortality 3

A total of 197 urinary samples were included for biomarker measurements. The median collection time for first and second urine sample was 11.2 (3.9, 27.9) hours and 19.5 (13.6, 37.7) hours post-AKI, respectively. The second urine sample was used for determining the predictive performance of various biomarkers. As shown in Table 3, levels of NGAL, TIMP-2, IGFBP-7 and CCL14 were significantly higher in children with persistent AKI, and levels of NGAL, IGFBP-7 and CCL4 were also significantly elevated in prolonged AKI.

**Table 3:** Urinary levels of biomarkers between children with and without persistent or prolonged acute kidney injury

Biomarker	No persistent AKI	Persistent AKI	*p*-value	No prolonged AKI	Prolonged AKI	*p*-value
NGAL (ng/ml) (***n*** = 82)	3.3 (1.4–11.1)	14.5 (4.6–64.7)	**0.001**	4.4 (1.9–12.9)	10.1 (4.7–34.5)	**0.024**
TIMP-2 (ng/ml) (***n*** = 79)	2.7 (0.6–5.9)	4.3 (1.9–6.9)	**0.038**	2.8 (0.7–6.1)	3.9 (2.8–6.2)	0.091
IGFBP-7 (ng/ml) (***n*** = 87)	22.1 (14.7–51.5)	42.1 (21.5–92.0)	**0.007**	23.8 (14.9–59.7)	40.4 (22.5–99.0)	**0.047**
TIMP-2*IGFBP-7 [(ng/ml)^2^/1000] (***n*** = 77)	0.5 (0.1–1.1)	1.0 (0.2–3.8)	0.074	0.6 (0.2–1.4)	1.0 (0.2–1.9)	0.274
CCL14 (pg/ml) (***n*** = 88)	163.7 (63.6–386.2)	396.9 (138.5–1144.7)	**0.004**	166.3 (77.1–424.3)	481.6 (124.8–1610.4)	**0.010**

Expressed as median (25^th^–75^th^ percentile)

The median (25^th^–75^th^) time of collecting the urine samples was 19.5 (13.6–37.7) hours after AKI

*AKI *acute kidney injury, *CCL14* C–C motif chemokine ligand 14, *CI *confidence interval, *IGFBP-7* insulin-like growth factor-binding protein 7, *NGAL* neutrophil gelatinase-associated lipocalin, *ROC* receiver operating characteristic, *TIMP-2* tissue metalloproteinases-2

We further explored the diagnostic performance of biomarkers across different models (Table 4 and Supplemental Table 3). The AKI staging of simultaneous SCr level for each urine sample was determined, and then each AKI stage was further stratified by biomarker positivity. The results revealed that all four biomarkers significantly predicted the development of persistent and prolonged AKI, with NGAL showing the best predictive performance. We found that incorporating simultaneous AKI staging into the biomarker measurements significantly enhanced the diagnostic performance of all biomarkers for both persistent and prolonged AKI. While the diagnostic performance of measuring multiple biomarkers in the same sample (AUC of 0.75 [0.64, 0.86] for persistent AKI and 0.75 [0.64,0.87] for prolonged AKI) and serial measurements of the same biomarkers also demonstrated acceptable prediction, they were comparable but not superior to single biomarker measurements. In addition, NGAL, TIMP-2 and IGFBP-7 positivity were significantly associated with the development of AKD (Supplemental Table 2).

**Table 4:** Predictive performance of urinary biomarkers for persistent and prolonged acute kidney injury

Biomarkers	Model	Persistent AKI		Prolonged AKI	
		AUC (95^th^ CI)	*p*-value	AUC (95^th^ CI)	*p*-value
NGAL	Biomarker positivity^	0.72 (0.61, 0.83)	**0.001**	0.72 (0.61, 0.84)	**0.004**
	Biomarker positivity and simultaneous SCr AKI staging	0.86 (0.78, 0.94)	**<0.001**	0.87 (0.79, 0.96)	**<0.001**
TIMP-2	Biomarker positivity^	0.65 (0.52, 0.78)	**0.003**	0.68 (0.54, 0.81)	**0.024**
	Biomarker positivity and simultaneous SCr AKI staging	0.85 (0.76, 0.93)	**<0.001**	0.87 (0.78, 0.96)	**<0.001**
IGFBP-7	Biomarker positivity^	0.67 (0.55, 0.78)	**0.010**	0.65 (0.52, 0.78)	**0.045**
	Biomarker positivity and simultaneous SCr AKI staging	0.86 (0.78, 0.94)	**<0.001**	0.86 (0.78, 0.95)	**<0.001**
CCL14	Biomarker positivity^	0.70 (0.58, 0.81)	**0.002**	0.69 (0.55, 0.83)	**0.008**
	Biomarker positivity and simultaneous SCr AKI staging	0.86 (0.77, 0.94)	**<0.001**	0.87 (0.78, 0.95)	**<0.001**
Serial biomarkers^#^	NGAL (*n*=59)	0.77 (0.65, 0.89)	**<0.001**	0.76 (0.64, 0.88)	**0.002**
	TIMP-2 (*n*=61)	0.72 (0.59, 0.85)	**0.003**	0.71 (0.57, 0.86)	**0.009**
	IGFBP-7 (*n*=64)	0.53 (0.39, 0.67)	0.705	0.57 (0.42, 0.71)	0.402
	CCL14 (*n*=66)	0.71 (0.64, 0.86)	**0.003﻿**	0.67 (0.51, 0.82)	**0.001**
Multiple biomarkers	Total number of +ve biomarkers^ (*n*=88)	0.75 (0.64, 0.86)	**<0.001**	0.75 (0.64, 0.87)	**<0.001**

*AKI* acute kidney injury, *AUC* area-under-curve, *CCL14* C-C motif chemokine ligand 14, *CI* confidence interval, *IGFBP-7* insulin-like growth factor-binding protein 7, *NGAL* neutrophil gelatinase-associated lipocalin, *SCr,* serum creatinine, *TIMP-2* tissue metalloproteinases-2

^The median (25^th^−75^th^ percentile) time of collecting the urine sample was 19.5 (13.6–37.7) hours after AKI, and -0.7 (-2.5 – 0.3) hours relative to the urine collection time for measuring the simultaneous serum creatinine level. The cut-off values for biomarker positivity: NGAL level ≥3.8 ng/ml for persistent AKI and ≥3.9 ng/ml for prolonged AKI; TIMP-2 level ≥3.1ng/ml for both persistent and prolonged AKI; IGFBP-7 level ≥24.3 ng/ml for both persistent AKI and prolonged AKI; CCL14 level ≥268.7 pg/ml for persistent AKI and ≥469.5 pg/ml for prolonged AKI

# The median (25^th^−75^th^ percentile) time of collecting the urine sample was 11.2 (3.9–27.9) hours after AKI for the 1 st sample and 19.5 (13.6–37.7) hours after AKI for the 2nd sample. The cut-off values for serial biomarker positivity: NGAL level ≥140.6 ng/ml (1^st^) and 3.75 ng/ml (2^nd^) for persistent AKI and 683.3 ng/ml (1^st^) and 3.9 ng/ml (2nd) for prolonged AKI; TIMP-2 level ≥18.6 ng/ml (1^st^) and 3.1 ng/ml (2^nd^) for persistent AKI and 20.6 ng/ml (1^st^) and 3.1 ng/ml (2^nd^) for prolonged AKI; IGFBP-7 level l ≥7.0 ng/ml (1^st^) and 24.3 ng/ml (2^nd^) for persistent AKI and 13.9 ng/ml (1^st^) and 24.3 ng/ml (2^nd^) for prolonged AKI; CCL14 level l ≥22752.9 pg/ml (1^st^) and 2268.7 pg/ml (2^nd^) for persistent AKI and 1226.7 pg/ml (1^st^) and 469.5 pg/ml (2^nd^) for prolonged AKI

## Discussion

We demonstrated that urinary NGAL, TIMP-2, IGFBP-7 and CCL14 exhibited satisfactory prediction of kidney function progression within 7 days of AKI onset in a diverse surgical and medical PICU population. In addition, combining the biomarker levels with simultaneous clinical information yielded a superior predictive performance. The findings refined our understanding of how to optimally apply biomarker measurement in clinical practice.

Among the four biomarkers evaluated in our cohort, NGAL showed the best predictive performance. Our findings for NGAL were consistent with two previous adult studies on persistent AKI prediction: The RUBY study reported an AUC of 0.71 (95% CI: 0.65–0.76) among 311 adults with severe AKI, and Lumlertgul et al. demonstrated an AUC of 0.75 (95% CI: 0.70–0.80) in 1322 adults within 3 days of AKI diagnosis [11, 23]. However, the optimal cutoff values for diagnosing persistent or prolonged AKI still require further evaluation as they varied across these studies and our data. We analyzed the performance of TIMP-2 and IGFBP-7 by measuring their levels using ELISA as the commercial point-of-care test was not available in our unit. Nevertheless, our result showed that both biomarkers were still moderately predictive of persistent and prolonged AKI: a finding comparable to the data obtained from studies employing the bedside point-of-care test [24, 25]. Our results supplemented and strengthened the applicability of using cell arrest biomarkers in the pediatric population. In addition to persistent AKI, we also demonstrated that NGAL, TIMP-2 and IGFBP-7 can predict both prolonged AKI and AKD. This offered important prognostic information as prolonged AKI implies progression to AKD, which is associated with long-term kidney function non-recovery.

The role of CCL14 in diagnosing persistent AKI has been evaluated in several studies among critically ill adults. A recent meta-analysis comprising 952 critically ill adults with AKI from 6 studies, reported a pooled sensitivity of 0.81 (95% CI 0.72–0.87) and specificity of 0.71 (95% CI 0.53–0.84) for urinary CCL14 in predicting persistent AKI [26]. To the best of our knowledge, this is the first report providing positive evidence for CCL14’s utility in pediatric patients. While a previous retrospective cohort study of children undergoing cardiac surgery found no association between urinary CCL14 and persistent severe AKI [27], this could be attributed to the limited sample size, the challenge of matching patients with different disease severity and variable urine collection times. Our positive findings could be explained by a larger sample size encompassing both medical and surgical patients, and the inclusion of all AKI stages as the outcome measure.

The timing of urine collection warranted close examination. Since SCr is a delayed marker, it is intuitive to expect that urine collected later in the course of AKI, when SCr has risen to indicate sustained kidney insult, might yield better diagnostic performance. However, the clinical importance of predicting persistent AKI also diminishes as the collecting time approaching 72 h post-AKI. In our cohort, the median time of urine collected was 19.5 h after AKI, which still allowed for outcome prediction in at least the subsequent 48 h. Nevertheless, we included AKI lasting for 7 days as an outcome, hoping to broaden the time window during which biomarkers could be applied in the PICU. The temporal changes in urinary concentrations following AKI have been extensively studied for NGAL, TIMP-2 and IGFBP-7 [13, 28]. Previous studies evaluating these 3 biomarkers reported a urine collection time within 6–72 h of AKI diagnosis [11, 23–25]. In contrast, Basu et al. showed a higher predictive performance with an AUC of 0.89 for persistent AKI using NGAL when urine was collected just 2 h after the initiation of cardiopulmonary bypass in pediatric patients [12]. In comparison, clinical data on the kinetics of CCL14 in AKI patients remain limited. In a cohort study of 48 patients with sepsis-associated AKI, Jiang et al. reported declining CCL14 levels measured at 6-h intervals over a 24-h period. However, the biomarker did not significantly predict persistent AKI [29]. Notably, this study did not employ the time of AKI onset as the reference point, which may have affected the interpretation of CCL14 dynamics. Similarly, the urine collection time in Chen et al.’s meta-analysis exhibited substantial variability, ranging from within one day of AKI diagnosis to just before the termination of kidney replacement therapy [26]. Given that CCL14 is postulated to be produced in injured renal tubules, a sufficient lag time may be necessary for its urinary concentration to become detectable. As such, the optimal timing for urinary CCL14 measurement remains unclear. Further studies will be required to determine the most effective urine collection window by serially measuring the urinary biomarker levels in relation to accurately defined AKI onset to delineate the time-concentration profile.

Integrating biomarker measurements with simultaneous clinical information significantly enhanced predictive performance. While the benefit of incorporating clinical risk factors into biomarker measurements has been well demonstrated in previous studies for AKI diagnosis [30, 31], our data further depicted that including simultaneous SCr levels can improve the prediction of AKI prognosis. This information can directly aid clinicians in refining management strategies: a more severe simultaneous SCr staging coupled with a positive biomarker reading signifies an increased risk of persistent or prolonged AKI. We employed the framework of revised AKI classification proposed at the 23rd Acute Disease Quality Initiative meeting, which incorporates biomarker positivity as a damage criterion into each AKI stage [15]. While acknowledging that biomarker measurement is not universally available, and that universally accepted cutoff values across diverse populations and settings are lacking, our results aligned with the consensus statement that this framework provides valuable prognostic insight. Further studies are required to develop and validate biomarker-guided clinical algorithms to support their broader implementation.

Our data demonstrated that both multiple and serial biomarker measurements exhibited satisfactory predictive performance, which were consistent with previous adult studies. Qian et al. reported an AUC of 0.77 (95% CI 0.70–0.83) for prediction of kidney function non-recovery using combined urinary CCL14 level and [TIMP-2] × [IGFBP-7] in 164 adult patients with AKI [32]. Similarly, a secondary analysis of the RUBY study by Prowle et al. showed that increasing and decreasing trends of CCL14 levels over a 12-h interval were associated with a higher and lower risk of persistent severe AKI, respectively, among 417 adults with severe AKI [33]. Our findings reinforced these observations, and further studies will certainly be required to validate these findings in different contexts. The non-invasive nature of serial urine sampling holds promise as a practical alternative to frequent blood sampling in selected clinical settings, serving as a valuable tool to refine the risk assessment and prognosis in AKI management.

Our study has several limitations. First, the estimation of incidence of persistent AKI, AKD and eGFR < 90 ml/min/1.73 m^2^ at 90 days may be subjected to bias, as the diagnosis was solely based on SCr criteria. We were unable to include urine output criteria for AKI diagnosis owing to incomplete data, which may have potentially underestimated the incidence of AKI and its associated outcomes [34]. However, our previous cohort study indicated that most of our children did not develop oliguria, and AKI was predominantly diagnosed using SCr criteria [35], suggesting that relying solely on SCr criteria still represented a relatively comprehensive cohort of children with AKI in our setting. Moreover, children with milder conditions may not have undergone follow-up blood testing. Second, incomplete urine sample collection resulted in an uneven number of samples both within and between individuals for different biomarkers, which certainly reduced the statistical power to compare diagnostic efficacy. Third, excluding children without urinary catheters may have excluded those with milder disease, potentially distorting the true diagnostic performance. Fourth, our results may have limited generalizability as this was a single-center study conducted in a tertiary referral hospital with a relatively small sample size. Nevertheless, our findings contribute to the growing understanding of the role of urinary biomarkers in predicting kidney function non-recovery in children. Incorporating urinary biomarker measurements into clinical care is potentially beneficial for informing prognosis, and we hope that our results can offer valuable insights into the development of biomarker-based algorithms.

## Conclusions

Critically ill children with AKI demonstrated a high incidence of persistent and prolonged AKI, conferring a substantial risk of long-term kidney dysfunction. Urinary NGAL, TIMP-2, IGFBP-7 and CCL14 demonstrated promising predictive capacity for kidney function non-recovery and can serve as a non-invasive prognosis-informing tool, and integrating urinary biomarkers with simultaneous clinical information yielded the strongest predictive performance. However, the optimal timing for biomarker measurement and cutoff values require further investigation before widespread clinical implementation can be recommended.

## Supplementary information

Below is the link to the electronic supplementary material.

Graphical abstract (PPTX 207 KB)

Supplementary file2 (DOCX 371 KB)

## Data Availability

The dataset generated is available from the corresponding author on reasonable request.
