# Supplementary material for: The use of urinary biomarkers as prognostic tools: predicting kidney outcomes in pediatric acute kidney injury
Source: Pediatr Nephrol. 2025 Aug 13;40(12):3815–23. doi: 10.1007/s00467-025-06920-0 (PMC12549750; doi:10.1007/s00467-025-06920-0)
Supplement: Supplementary file 1 — Graphical abstract (PPTX 207 KB) [file 467_2025_6920_MOESM1_ESM.pptx]

## Slide 1
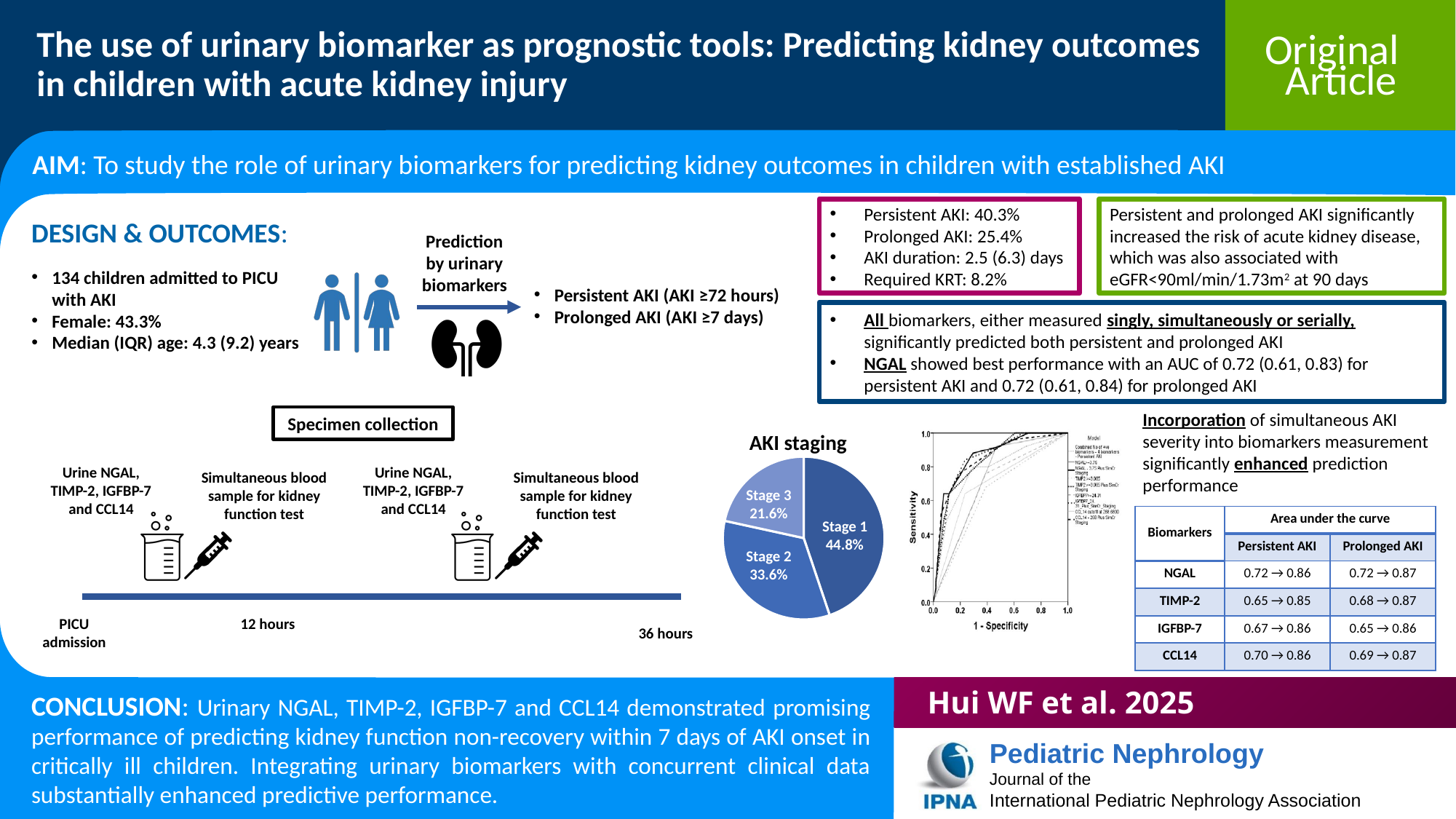

The use of urinary biomarker as prognostic tools: Predicting kidney outcomes in children with acute kidney injury
AIM: To study the role of urinary biomarkers for predicting kidney outcomes in children with established AKI
Persistent AKI: 40.3%
Prolonged AKI: 25.4%
AKI duration: 2.5 (6.3) days
Required KRT: 8.2%
Persistent and prolonged AKI significantly increased the risk of acute kidney disease, which was also associated with eGFR<90ml/min/1.73m2 at 90 days
DESIGN & OUTCOMES:
Prediction by urinary biomarkers
134 children admitted to PICU with AKI
Female: 43.3%
Median (IQR) age: 4.3 (9.2) years
Persistent AKI (AKI ≥72 hours)
Prolonged AKI (AKI ≥7 days)
All biomarkers, either measured singly, simultaneously or serially, significantly predicted both persistent and prolonged AKI
NGAL showed best performance with an AUC of 0.72 (0.61, 0.83) for persistent AKI and 0.72 (0.61, 0.84) for prolonged AKI
Incorporation of simultaneous AKI severity into biomarkers measurement significantly enhanced prediction performance
Specimen collection
AKI staging
### Chart
| Category | Sales |
|---|---|
| Stage 1 | 44.8 |
| Stage 2 | 33.6 |
| Stage 3 | 21.6 |Stage 3 21.6%
Stage 1 44.8%
Stage 2 33.6%
Urine NGAL, TIMP-2, IGFBP-7 and CCL14
Urine NGAL, TIMP-2, IGFBP-7 and CCL14
Simultaneous blood sample for kidney function test
Simultaneous blood sample for kidney function test
PICU admission
12 hours
36 hours
| Biomarkers | Area under the curve | |
| --- | --- | --- |
| | Persistent AKI | Prolonged AKI |
| NGAL | 0.72 → 0.86 | 0.72 → 0.87 |
| TIMP-2 | 0.65 → 0.85 | 0.68 → 0.87 |
| IGFBP-7 | 0.67 → 0.86 | 0.65 → 0.86 |
| CCL14 | 0.70 → 0.86 | 0.69 → 0.87 |
Hui WF et al. 2025
CONCLUSION: Urinary NGAL, TIMP-2, IGFBP-7 and CCL14 demonstrated promising performance of predicting kidney function non-recovery within 7 days of AKI onset in critically ill children. Integrating urinary biomarkers with concurrent clinical data substantially enhanced predictive performance.
