# Supplementary material for: The use of urinary biomarkers as prognostic tools: predicting kidney outcomes in pediatric acute kidney injury
Source: Pediatr Nephrol. 2025 Aug 13;40(12):3815–23. doi: 10.1007/s00467-025-06920-0 (PMC12549750; doi:10.1007/s00467-025-06920-0)
Supplement: Supplementary file 2 — Supplementary file2 (DOCX 371 KB) [file 467_2025_6920_MOESM2_ESM.pdf]

Supplementary materials

Table 1: Diagnostics criteria and definition
Table 2: Risk factors for development of acute kidney disease
Table 3: Diagnostic performance of biomarkers for persistent and prolonged acute kidney injury
Figure 1: Consort diagram
Figure 2: Probability of eGFR > 90ml/min/1.73m^2 after 90 days post-AKI

Supplementary table 1: Diagnostics criteria and definition

**Diagnosis of AKI**	• Increase in serum creatinine level (SCr) by ≥26.5 umol/L ≤ 48 hours OR<br>• Increase in SCr to ≥ 1.5 times of baseline, which is known or presumed to have occurred within the prior 7days
**Persistent AKI**	AKI lasting consecutively for ≥ 72 hours
**Diagnosis of AKD**	Children who fulfilled SCr criteria from 7 days after diagnosing AKI till 90 days

Staging of AKI and AKD

Stage	AKI	AKD
**Stage 1**	• SCr 1.5-1.9 times of baseline OR<br>• ≥ 26.5umol/L increase	SCr 1.5-1.9 times of baseline
**Stage 2**	SCr 2.0-2.9 times of baseline	SCr 2.0-2.9 times of baseline
**Stage 3**	• ≥3.0 times of baseline OR<br>• Increase in SCr to ≥ 353.6umol/L OR<br>• Initiation of renal replacement therapy OR<br>• Decrease in eGFR < 35ml/min/1.73m^2 if < 18 years	• SCr ≥ 3.0 times of baseline OR<br>• Ongoing renal replacement therapy

AKD: acute kidney disease; AKI: acute kidney injury; eGFR: estimated glomerular filtration rate; SCr: serum creatinine; UO: urine output

Supplemental table 2: Risk factors for development of acute kidney disease

Variable		Unadjusted HR (95% CI)	p-value	Adjusted HR (95% CI)	p-value
Female sex		1.265 (0.768, 2.083)	0.356	---	---
Age (year)		0.988 (0.942, 1.036)	0.621	---	---
Post-cardiac surgery		1.334 (0.754, 2.359)	0.322	---	---
History of malignancy		1.194 (0.717, 1.989)	0.495	---	---
BMT recipient		1.245 (0.447, 3.468)	0.675	---	---
PIM3 predicted mortality (%)		1.012 (0.964, 1.062)	0.623	---	---
Severe AKI (Stage 2 or stage 3 AKI)		1.735 (1.026, 2.936)	**0.040**	0.884 (0.188, 4.167)	0.876
Persistent AKI		2.592 (1.548, 4.340)	**<0.001**	---	**---**
Prolonged AKI		2.560 (1.560, 4.201)	**<0.001**	---	
Duration of AKI (day)		1.208 (1.100, 1.327)	**<0.001**	1.302 (0.954, 1.776)	0.096
Required kidney replacement therapy		1.812 (0.768, 4.273)	0.175	---	---
Required inotropes		0.979 (0.563, 1.704)	0.941	---	
Duration of inotropes use (day)		1.004 (0.991, 1.018)	0.516	---	---
Required mechanical ventilation		0.957 (0.554, 1.653)	0.875	---	
Duration of mechanical ventilation (day)		1.001 (0.992, 1.010)	0.840	---	---
Required non-invasive ventilation		0.759 (0.421, 1.366)	0.358	---	
Duration of non-invasive ventilation (day)		0.996 (0.986, 1.007)	0.507	---	---
Biomarker positivity	NGAL	1.999 (1.041, 3.838)	**0.037**	---	---
	TIMP-2	2.571 (1.326, 4.988)	**0.005**	---	---
	IGFBP-7	2.625 (1.418, 4.860)	**0.002**	---	---
	CCL14	1.578 (0.853, 2.920)	0.146	---	
Total number of +ve biomarkers		1.459 (1.166, 1.826)	**0.001**	1.097 (0.655, 1.836)	0.725

AKI: acute kidney injury; BMT: bone marrow transplantation; CCL14: C-C motif chemokine ligand 14; CI: confidence interval; HR: hazard ratio; IGFBP-7: insulin-like growth factor-binding protein 7; NGAL: neutrophil gelatinase-associated lipocalin; PIM3: pediatric index of mortality 3; TIMP-2: tissue metalloproteinases-2.

Supplemental table 3: Diagnostic performance of biomarkers for persistent and prolonged acute kidney injury

Model			Relative risk (95% CI)		Positive predictive value (%)		Negative predictive value (%)	
			Persistent AKI	Prolonged AKI	Persistent AKI	Prolonged AKI	Persistent AKI	Prolonged AKI
Urine NGAL - Biomarker +ve^			5.47 (1.81-16.54)	11.5 (1.6-82.1)	56.8 (49.0-64.4) %	38.8 (32.8-45.3) %	88.9 (72.6-96.0) %	96.5 (80.0-99.5) %
Urine NGAL^ and simultaneous SCr AKI staging	Stage 3 AKI	Biomarker +ve	2.37 (1.38-4.06)	4.11 (2.18-7.72)	82.4 (35.4-97.6) %	81.9 (34.9-97.4) %	62.6 (59.2-66.0) %	78.6 (74.3-82.2) %
		Biomarker -ve	2.79 (2.09-3.74)	4.50 (2.99-6.76)	100 (2.5-100) %	100 (2.5-100) %	60.5 (58.9-62.1) %	75.6 (73.6-77.5) %
	Stage 2 AKI	Biomarker +ve	3.35 (2.07-5.40)	3.71 (1.81-7.60)	84.5 (63.1-94.6) %	59.2 (38.4-77.2) %	72.4 (65.0-78.6) %	83.2 (76.2-88.5) %
		Biomarker -ve	0.44 (0.03-5.65)	0.51 (0.04-7.05)	---	---	58.8 (57.4-60.1) %	73.7 (72.6-74.7) %
	Stage 1 AKI	Biomarker +ve	1.26 (0.66-2.40)	1.10 (0.42-2.87)	49.4 (28.9-70.0) %	27.3 (12.1-50.8) %	62.0 (56.1-67.7) %	75.1 (69.9-79.6) %
		Biomarker -ve	0.38 (0.10-1.40)	0.13 (0.01-2.00)	18.6 (5.2-48.9) %	---	55.7 (51.4-60.0) %	70.0 (67.3-72.6) %
	No AKI	Biomarker +ve	0.38 (0.10-1.40)	0.29 (0.04-2.02)	18.6 (5.2-48.9) %	8.6 (1.3-40.4) %	55.7 (51.4-60.0) %	71.5 (68.1-74.7) %
		Biomarker -ve	0.07 (0.00-1.08)	0.11 (0.01-1.71)	---	---	51.3 (47.0-55.6) %	69.1 (66.1-72.0) %
Serial NGAL[#]	Both biomarkers +ve		2.18 (1.35-3.52)	3.17 (1.52-6.64)	77.4 (44.3-93.7) %	73.3 (23.5-96.1) %	66.8 (60.5-72.5) %	77.9 (75.5-81.8) %
Urine TIMP-2 - Biomarker +ve^			2.32 (1.15-4.66)	3.41 (1.23-9.46)	54.1 (43.8-64.0) %	38.3 (29.8-47.7) %	74.9 (61.5-84.8) %	88.4 (75.7-94.9) %
Urine TIMP-2^ and simultaneous SCr AKI staging	Stage 3 AKI	Biomarker +ve	2.34 (1.22-4.51)	3.75 (1.82-7.74)	79.6 (30.0-97.3) %	77.6 (27.7-96.9) %	62.0 (58.7-65.3) %	77.6 (73.8-81.0) %
		Biomarker -ve	3.00 (2.19-4.11)	4.59 (3.01-6.99)	100 (2.5-100) %	100 (2.5-100) %	60.6 (58.8-62.4) %	75.7 (73.6-77.7) %
	Stage 2 AKI	Biomarker +ve	2.99 (1.80-4.96)	4.06 (1.99-8.30)	81.3 (56.5-93.5) %	64.9 (40.8-83.2) %	68.9 (62.2-74.9) %	82.9 (76.1-88.1) %
		Biomarker -ve	2.12 (1.09-4.09)	1.52 (0.45-5.11)	72.2 (33.7-93.0) %	36.6 (10.3-74.3) %	62.6 (58.6-66.4) %	75.5 (72.1-78.7) %
	Stage 1 AKI	Biomarker +ve	0.74 (0.30-1.83)	0.53 (0.14-2.07)	32.1 (14.3-57.4) %	15.1 (4.2-41.7) %	57.8 (52.6-62.9) %	72.2 (67.8-76.2) %
		Biomarker -ve	0.70 (0.25-1.96)	0.33 (0.05-2.24)	30.2 (11.3-59.5) %	9.5 (1.4-43.1) %	58.0 (53.5-62.3) %	71.8 (68.4-75.0) %
	No AKI	Biomarker +ve	0.71 (0.21-3.46)	0.52 (0.08-3.42)	30.2 (8.6-66.7) %	14.2 (2.1-55.6) %	58.6 (55.0-62.1) %	73.4 (70.576.1) %
		Biomarker -ve	0.05 (0.00-0.81)	0.08 (0.00-1.23)	---	---	47.7 (42.4-53.1) %	66.4 (62.4-70.2) %
Serial TIMP-2[#]	Both biomarkers +ve		2.42 (1.78-3.28)	3.69 (2.43-5.60)	100 (29.2-100) %	100 (15.8-100) %	62.5 (59.3-65.6) %	76.8 (73.7-79.6) %
Urine IGFBP-7 – Biomarker +ve^			2.55 (1.29-5.04)	2.72 (1.09-6.78)	54.8 (45.5-63.7) %	35.6 (28.0-44.0) %	77.5 (64.5-86.7) %	86.7 (74.7-93.6) %
Urine IGFBP-7^ and simultaneous SCr AKI staging	Stage 3 AKI	Biomarker +ve	2.50 (1.56-4.01)	4.22 (2.39-7.43)	85.3 (41.5-97.9) %	84.3 (39.8-97.7) %	63.3 (60.0-66.8) %	79.2 (74.9-82.9) %
		Biomarker -ve	2.77 (2.09-3.67)	4.30 (2.93-6.31)	100 (2.5-100) %	100 (2.5-100) %	60.5 (59.0-61.9) %	75.5 (73.7-77.2) %
	Stage 2 AKI	Biomarker +ve	2.66 (1.67-4.25)	2.22 (1.07-4.59)	77.7 (55.1-90.8) %	45.4 (26.1-66.2) %	68.7 (62.5-74.4) %	79.2 (73.5-83.9) %
		Biomarker -ve	2.15 (1.13-4.06)	3.46 (1.72-6.95)	77.7 (27.4-97.0) %	76.3 (26.1-96.7) %	61.6 (58.8-64.3) %	77.1 (73.9-80.1) %
	Stage 1 AKI	Biomarker +ve	1.16 (0.55-2.41)	1.04 (0.36-3.01)	45.3 (22.3-70.6) %	26.3 (9.6-54.5) %	60.5 (56.1-64.7) %	74.7 (70.8-78.3) %
		Biomarker -ve	0.55 (0.22-1.36)	0.19 (0.03-1.33)	24.9 (10.7-48.0) %	5.9 (0.9-30.8) %	55.8 (50.8-60.7) %	69.6 (65.9-73.1) %
	No AKI	Biomarker +ve	0.38 (0.10-1.40)	0.28 (0.04-1.94)	17.4 (4.8-47.2) %	8.2 (1.2-39.2) %	55.8 (51.9-59.7) %	71.6 (68.5-74.5) %
		Biomarker -ve	0.06 (0.00-0.94)	0.09 (0.01-1.44)	---	---	50.6 (46.2-55.0) %	68.6 (65.4-71.5) %
Serial IGFBP-7[#]	Both biomarkers +ve		NA	NA	NA	NA	NA	NA
Urine CCL14 – Biomarker +ve^			2.76 (1.53-4.96)	3.39 (1.70-6.77)	62.3 (50.2-73.0) %	52.7 (36.5-68.3) %	76.4 (66.0-84.3) %	84.3 (77.1-89.6) %
Urine CCL14^ and simultaneous SCr AKI staging	Stage 3 AKI	Biomarker +ve	2.44 (1.53-3.89)	4.88 (3.20-7.46)	84.9 (40.7-97.9) %	100 (47.8-100) %	63.2 (59.6-66.5) %	79.2 (75.2-82.7) %
		Biomarker -ve	2.72 (2.06-3.58)	2.05 (0.49-8.60)	100 (2.5-100) %	50.5 (6.3-94.0) %	60.4 (59.0-61.9) %	75.2 (73.4-76.9) %
	Stage 2 AKI	Biomarker +ve	3.02 (1.98-4.60)	3.04 (1.51-6.10)	87.1 (61.7-96.6) %	67.1 (28.6-91.2) %	69.2 (63.3-74.5) %	77.8 (74.0-81.0) %
		Biomarker -ve	1.37 (0.58-3.20)	1.98 (0.94-4.17)	52.9 (19.4-84.0) %	43.4 (22.3-66.3) %	60.6 (57.6-63.6) %	78.0 (73.0-82.3) %
	Stage 1 AKI	Biomarker +ve	1.21 (0.55-2.66)	1.37 (0.41-4.51)	47.4 (20.6-75.7) %	33.8 (9.1-72.2) %	60.5 (56.8-64.1) %	75.2 (72.4-77.8) %
		Biomarker -ve	0.67 (0.32-1.40	0.40 (0.13-1.23)	29.7 (15.5-49.3) %	12.2 (4.4-29.6) %	56.2 (50.4-61.9) %	69.4 (64.1-74.2) %
	No AKI	Biomarker +ve	0.31 (0.05-1.99)	0.65 (0.10-4.04)	13.9 (2.0-55.5) %	17.0 (2.5-62.3) %	57.1 (54.2-60.0) %	74.0 (71.7-76.1) %
		Biomarker -ve	0.09 (0.01-0.65)	0.06 (0.00-0.92)	5.1 (0.8-27.5) %	---	48.6 (43.2-54.9) %	65.1 (60.9-69.2) %
Serial CCL14[#]	Both biomarkers +ve		2.20 (1.42-3.41)	2.59 (1.28-5.24)	83.8 (39.7-97.6) %	58.4 (27.1-84.1) %	64.5 (60.0-68.8) %	78.9 (73.8-83.1) %
Total number of +ve biomarkers (n=88)			1.50 (1.22-1.84)	1.71 (1.29-2.27)	73.0 (51.1-87.5) %*	60.5 (32.2-83.2) %*	68.0 (61.7-73.5) %*	79.1 (74.5-83.2) %*

AKI: acute kidney injury; AUC: area-under-curve; CCL14: C-C motif chemokine ligand 14; CI: confidence interval; IGFBP-7: insulin-like growth factor-binding protein 7; NA: Not available; NGAL: neutrophil gelatinase-associated lipocalin; SCr: serum creatinine; TIMP-2: tissue metalloproteinases-2

^The median (25th-75th percentile) time of collecting the urine sample was 19.5 (13.6 – 37.7) hours after AKI, and -0.7 (-2.5 – 0.3) hours relative to the urine collection time for measuring the simultaneous serum creatinine level. The cut-off values for biomarker positivity: NGAL level≥3.8ng/ml for persistent AKI and ≥3.9ng/ml for AKI=7 days; TIMP-2 level≥3.1ng/ml for both persistent AKI and AKI=7 days; IGFBP-7 level≥24.3ng/ml for both persistent AKI and AKI=7 days; CCL14 level ≥268.7pg/ml for persistent AKI and ≥469.5pg/ml for AKI=7 days

#The median (25th-75th percentile) time of collecting the urine sample was 11.2 (3.9 -27.9) hours after AKI for the 1st sample and 19.5 (13.6 – 37.7) hours after AKI for the 2nd sample. The cut-off values for serial biomarker positivity: NGAL level≥140.6ng/ml (1st) and 3.75ng/ml (2nd) for persistent AKI and 683.3ng/ml (1st) and 3.9ng/ml (2nd) for AKI=7days; TIMP-2 level≥18.6ng/ml (1st) and 3.1ng/ml (2nd) for persistent AKI and 20.6ng/ml (1st) and 3.1ng/ml (2nd) for AKI=7days; IGFBP-7 level l≥7.0ng/ml (1st) and 24.3ng/ml (2nd) for persistent AKI and 13.9ng/ml (1st) and 24.3ng/ml (2nd) for AKI=7days; CCL14 level l≥22752.9pg/ml (1st) and 2268.7pg/ml (2nd) for persistent AKI and 1226.7pg/ml (1st) and 469.5pg/ml (2nd) for AKI=7days.

*All four biomarkers were positive

Supplemental figure 1: Consort diagram

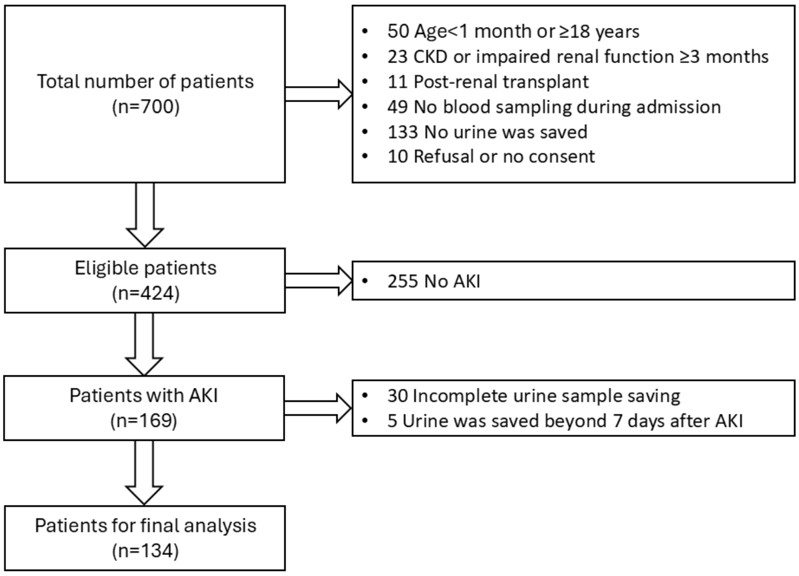

AKI: acute kidney injury; CKD: chronic kidney disease

Supplemental figure 2: Probability of eGFR > 90ml/min/1.73m^2 after 90 days post-AKI

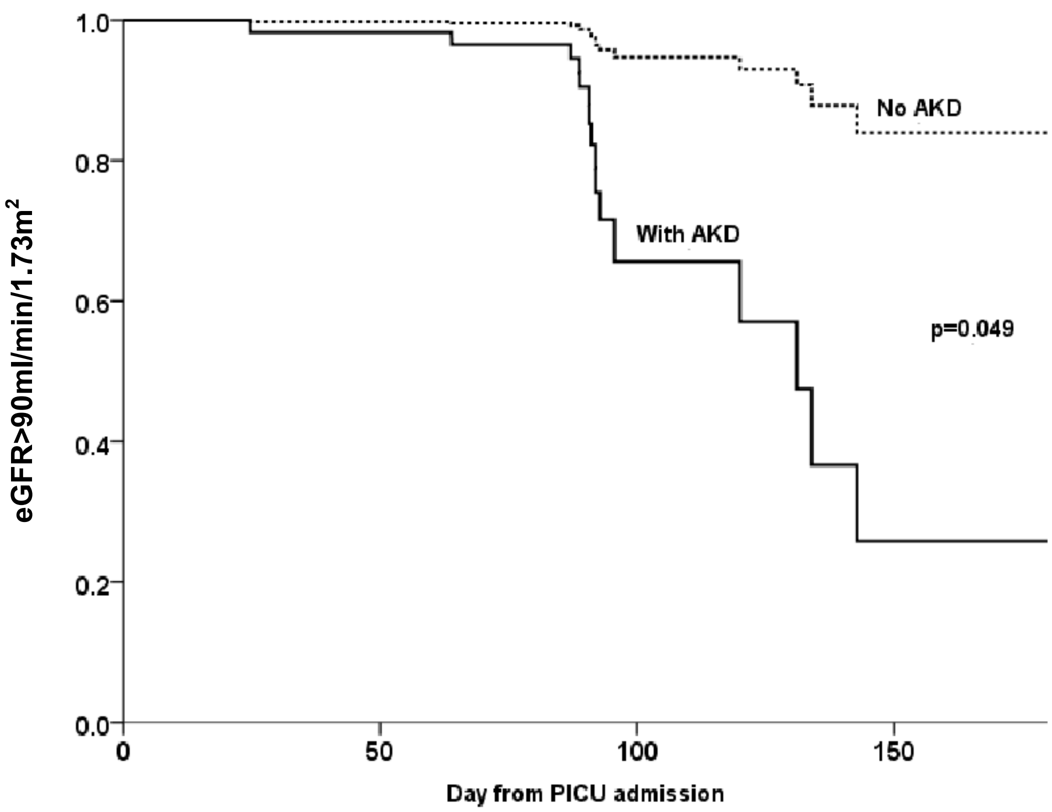

AKD: acute kidney disease; AKI: acute kidney injury; eGFR: estimated glomerular filtration rate; PICU: pediatric intensive care unit

*The eGFR was determined by the first available renal function test 90 days after acquiring AKI
